# A Roadmap for Tick-Borne Flavivirus Research in the “Omics” Era

**DOI:** 10.3389/fcimb.2017.00519

**Published:** 2017-12-22

**Authors:** Jeffrey M. Grabowski, Catherine A. Hill

**Affiliations:** ^1^Biology of Vector-Borne Viruses Section, Laboratory of Virology, Rocky Mountain Laboratories, National Institutes of Allergy and Infectious Diseases, National Institutes of Health, Hamilton, MT, United States; ^2^Department of Entomology, Purdue University, West Lafayette, IN, United States; ^3^Purdue Institute of Inflammation, Immunology and Infectious Disease, Purdue University, West Lafayette, IN, United States

**Keywords:** tick-borne flavivirus, *Flaviviridae*, *Ixodidae*, genomics, genetics, vaccine, anti-viral, acaricide

## Abstract

Tick-borne flaviviruses (TBFs) affect human health globally. Human vaccines provide protection against some TBFs, and antivirals are available, yet TBF-specific control strategies are limited. Advances in genomics offer hope to understand the viral complement transmitted by ticks, and to develop disruptive, data-driven technologies for virus detection, treatment, and control. The genome assemblies of *Ixodes scapularis*, the North American tick vector of the TBF, Powassan virus, and other tick vectors, are providing insights into tick biology and pathogen transmission and serve as nucleation points for expanded genomic research. Systems biology has yielded insights to the response of tick cells to viral infection at the transcript and protein level, and new protein targets for vaccines to limit virus transmission. Reverse vaccinology approaches have moved candidate tick antigenic epitopes into vaccine development pipelines. Traditional drug and *in silico* screening have identified candidate antivirals, and target-based approaches have been developed to identify novel acaricides. Yet, additional genomic resources are required to expand TBF research. Priorities include genome assemblies for tick vectors, “omic” studies involving high consequence pathogens and vectors, and emphasizing viral metagenomics, tick-virus metabolomics, and structural genomics of TBF and tick proteins. Also required are resources for forward genetics, including the development of tick strains with quantifiable traits, genetic markers and linkage maps. Here we review the current state of genomic research on ticks and tick-borne viruses with an emphasis on TBFs. We outline an ambitious 10-year roadmap for research in the “omics era,” and explore key milestones needed to accomplish the goal of delivering three new vaccines, antivirals and acaricides for TBF control by 2030.

## Introduction

Ticks (subphylum Chelicerata, subclass Acari, suborder Ixodida) are ectoparasites of humans and animals, and vectors of bacteria, protozoa, and viruses (Gulia-Nuss et al., 2016). Scientists have documented more than 38 species of viruses comprising members of the families Asfarviridae, Reoviridae, Rhabdoviridae, Orthomyxoviridae, Bunyaviridae, Flaviviridae, and possibly the Arenaviridae that are transmitted by ticks (Labuda and Nuttall, 2004). Reports of tick-borne viruses are increasing (Mansfield et al., 2017a,b), and new viruses are emerging such as the Heartland and Bourbon viruses identified in the U.S.A. (https://www.cdc.gov/heartland-virus/index.html; https://www.cdc.gov/ncezid/dvbd/bourbon/index.html). The geographic ranges of tick species are expanding (Medlock et al., 2013), yet the implications for disease epidemiology are not well understood. There is growing appreciation of the complexity of the tick “microbiome,” defined as the complement of pathogens, commensals and symbionts carried in or on a tick, variation at spatial and temporal scale (Narasimhan and Fikrig, 2015; Van Treuren et al., 2015), and the prevalence of tick co-infections (Diuk-Wasser et al., 2016; Moutailler et al., 2016). Knowledge regarding virus species transmitted by ticks is limited, and many pathogenic viruses may go unnoticed or undiagnosed (Hubalek and Rudolf, 2012; Lani et al., 2014). Research is now pivoting to determine the complement of viruses acquired by ticks during blood feeding, and the role of these viruses in pathogenesis, with data-driven research likely to facilitate a precision medicine approach to the diagnosis and treatment of TBFs.

Of the viruses transmitted by ticks, the tick-borne flaviviruses (TBFs) are considered the most important affecting human health globally. TBFs are transmitted by multiple species of ixodid ticks in the families Ixodidae (hard ticks) and Argasidae (soft ticks) (Table 1). In the past two decades there has been a notable increase in the incidence of TBF disease (Lasala and Holbrook, 2010). Among TBFs, tick-borne encephalitis virus (TBEV) is regarded as one of the most dangerous human neuroinfections in Europe and Asia where it causes between 10,000 and 15,000 human cases every year, respectively (Gritsun et al., 2003a,b; Dobler, 2010; Rumyantsev et al., 2013). Other members of the TBF complex of importance to public health include Louping-ill virus (LIV) in the United Kingdom, Omsk hemorrhagic fever virus (OHFV) in parts of Russia, Kyasanur Forest Disease virus (KFDV) in parts of India, Alkhurma hemorrhagic fever virus (AHFV) in Saudi Arabia, and Powassan encephalitis virus (POWV), including deer tick virus Powassan lineage II, the only human pathogenic TBF detected in North America to date (Dobler, 2010).

**Table 1:** Summary of tick-borne flaviviruses associated with disease in humans, geographic location, proposed tick vectors and vaccine approaches.

**TBF/Subtypes**	**Species of ticks that serve as potential vectors**	**Estimated no. human cases per annum**	**Geographic distribution**	**Vaccine(s) available**	**Biosafety level in USA**
Kyasanur Forest Disease Virus (KFDV)	*Hemaphysalis spinigera Hemaphysalis turturis*	400–500^a^	India	No^h^	4
Alkhurma Hemorrhagic Fever Virus (AHFV)	*Ornithodoros savignyi Hyalomma dromedari*	77^b^	Arabian Peninsula	No	4
Omsk Hemorrhagic Fever Virus (OHFV)	*Dermacentor reticulatus Dermacentor marginatus Ixodes persulcatus Ixodes apronophorus*	24^c^	Russia	No	4
Tick-Borne Encephalitis Virus (TBEV) European/Western Siberian Far-eastern	*Hemaphysalis concinna Hemaphysalis punctata Dermacentor reticulatus Ixodes ricinus Ixodes persulcatus*	10,000-15,000^d^	Europe and Asia	Yes	4
Powassan Virus (POWV)	*Haemaphysalis longicornis Dermacentor andersoni Ixodes marxi Ixodes cookei Ixodes scapularis (for POWV lineage II: deer tick virus)*	5^e^	North America and Russia	No	3
Louping Ill Virus (LIV)	*Ixodes ricinus*	No confirmed cases since early 1990s^f^	United Kingdom	No	3
Langat Virus (LGTV)	*Ixodes granulatus Hemaphysalis papuana*	Unknown; cases recorded only during use of virus in anti-TBEV vaccination trials^g^	Southeast Asia	No	2

*Kyasanur Forest disease virus, KFDV; Alkhurma hemorrhagic fever virus, AHFV; Omsk hemorrhagic fever virus, OHFV; Tick-borne encephalitis virus, TBEV; Powassan virus, POWV; Louping ill virus, LIV; Langat virus, LGTV*.

a *(Holbrook, 2012; Kasabi et al., 2013; Lani et al., 2014; Grabowski et al., 2016)*.

b *Average from 2009–2011 (Alzahrani et al., 2010; Memish et al., 2014)*.

c *Average from 1946–2000 (Gritsun et al., 2003a; Grabowski et al., 2016)*.

d *(Mansfield et al., 2009; Dobler, 2010)*.

e *Average from 2000–2013 in USA (Paddock et al., 2016)*.

f *(Jeffries et al., 2014)*.

g *(Gritsun et al., 2003a,b)*.

h *A vaccine is licensed and available in endemic areas of India (Holbrook, 2012; Kasabi et al., 2013)*.

Next generation sequencing (NGS) technologies have allowed the generation of new resources for tick-borne disease research. Genomics has enabled reverse genetics to identify tick proteins and biochemical pathways that could be targeted to disrupt virus transmission. The assembly of the *Ixodes scapularis* (black-legged tick) genome (Gulia-Nuss et al., 2016), a vector of POWV, is the first such resource for a tick and a nucleation point for tick genome research. Draft genome assemblies are available for the castor bean tick, *Ixodes ricinus* (Cramaro et al., 2015), also a TBF vector, and for the southern cattle tick, *Rhipicephalus* (*Boophilus*) *microplus* (Guerrero et al., 2006, 2010; Barrero et al., 2017). These resources will enable investigations of tick-pathogen relationships in a “genome-wide” context and comparative genomic research between lineages comprising major tick vectors. Progress in gene discovery for species of hard and soft ticks has been extensive (Meyer and Hill, 2014), with an emphasis on elucidating gene products associated with tick-host-pathogen interactions. Whole genome computational analyses have revealed duplication events involving large numbers of genes in *I. scapularis* and other species of hard ticks that may be associated with the evolution of parasitic strategies (Van Zee et al., 2016). Transcriptome and proteome studies have examined the molecular response of *Ixodes* cells to viral infection (Villar et al., 2015; Weisheit et al., 2015; Grabowski et al., 2016; Mansfield et al., 2017a) and functional analyses have investigated proteins that exhibited differential expression post infection with virus (Schnettler et al., 2014; Ayllon et al., 2015a; Weisheit et al., 2015; Grabowski et al., 2017a).

Despite these achievements, there remain challenges to the identification of protein targets for vaccine, drug, and acaricide development. Deliberate investment in resources for forward and reverse genetics with an emphasis on major tick vectors and pathogenic virus strains is required. Metabolomics and structural genomics represent new frontiers. When coupled with sequence-based genetic mapping and tools for genetic transformation, these fields have the potential to identify molecular targets and guide the rational design of transmission blocking vaccines and acaricides. The scope of genomic resources required is substantial given the biological complexities of TBF transmission. Here we present a 10-year roadmap for research to expand the arsenal of TBF control technologies and deliver three new antiviral, vaccine, and acaricide products by a proposed target date of 2030. The roadmap and associated milestones are intended as a framework to guide discussions between the research community and funding agencies. While ambitious, the importance of TBFs necessitates commitment to strategic research priorities to ensure the timely achievement of public health goals.

### Tick-borne flaviviruses

TBFs are enveloped, positive-strand RNA viruses in the family Flaviviridae that includes dengue (DENV), hepatitis C (HCV), Japanese encephalitis (JEV), West Nile (WNV), and Zika (ZIKV) viruses. Many TBFs cause significant human and animal disease worldwide (Table 1) and are transmitted primarily via the bite of an infected tick. In nature, TBFs are maintained in a cycle between small mammal reservoirs and ticks. However, the complex transmission cycles of many TBFs have not been resolved and studies to incriminate tick species in virus transmission are needed. Most TBFs are classified Biosafety-level (BSL) 3 and 4 (Table 1). In humans, symptoms of TBF infection range from febrile illness to more serious encephalitis and hemorrhagic complications. Case fatality rates as high as 20% have been recorded for the most pathogenic TBFs (e.g., far-eastern form of TBEV). Multiple vaccines are available in Europe for TBEV, although no TBF-specific antivirals or transmission-blocking vaccines have been developed. At present, TBF treatment and prevention options are considered lacking (Lani et al., 2014).

The focus of tick-borne disease research is shifting from a “one pathogen-one disease” mindset toward an understanding of disease in the context of the “pathobiome” (Vayssier-Taussat et al., 2015). Genomic studies have emphasized high consequence pathogens and their impact on the human host, as well as flavivirus biodiversity and evolution, but there is need to determine the complement of virus species that circulate in host and reservoir populations. NGS involving 454 and Illumina-based 16S rRNA pyrosequencing has been used to explore bacterial communities associated with *I. ricinus* (Carpi et al., 2011), *I. scapularis* (Van Treuren et al., 2015), and *Amblyomma americanum* (Ponnusamy et al., 2014; Williams-Newkirk et al., 2014; Trout Fryxell and DeBruyn, 2016). Pyrosequencing of DNA enriched for bacteria/arachaea has also been used to evaluate the microbiome of seven hard tick species (Nakao et al., 2013). RNAseq revealed that the Flaviviridae infect a wider range of invertebrate hosts and exhibit greater diversity in genome structure than previously anticipated (Shi et al., 2015), but the implications for pathogenesis remain unclear. The relatively small size of TBF genomes (~10–15 Kb) makes viral whole genome sequencing (WGS) feasible. Future studies must emphasize viral metagenomics using WGS to define the viral phyla associated with ticks (Brinkmann et al., 2016). Information from these studies will guide the development of comprehensive, region-specific molecular diagnostic tools and healthcare guidelines.

**Figure 1:**
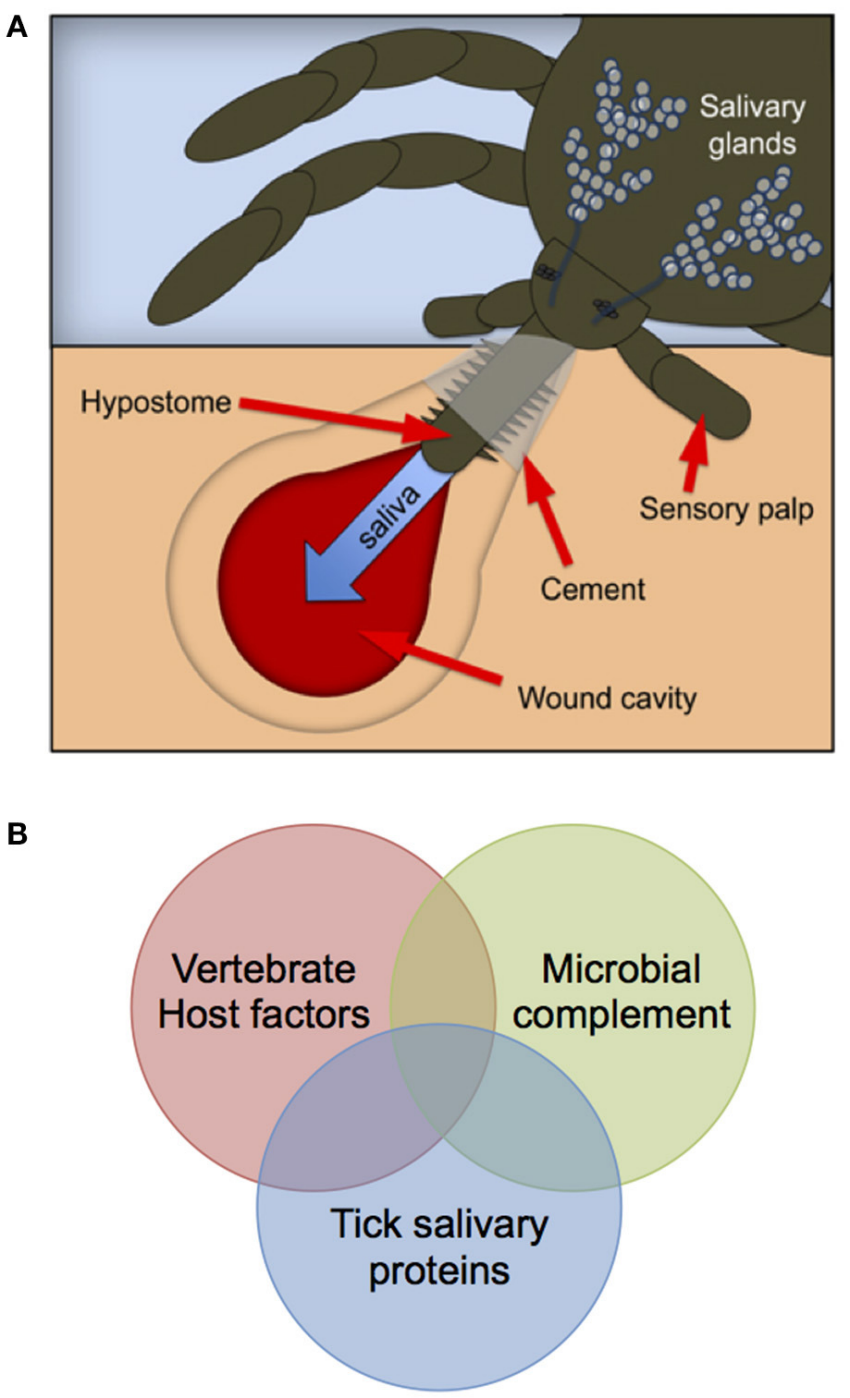
Schematic depicting the concept of the “systems biology” of a tick bite. Each tick bite **(A)** comprises a unique combination of host-derived factors, tick salivary proteins and the microbial flora delivered to the feeding site **(B)**, thus underpinning the need for “personalized” approaches to pathogen detection and treatment. **(A)** reproduced from Figure 1 of Gulia-Nuss et al. (2016) and reprinted by permission from Macmillan Publishers Ltd. ©copyright 2016.

De-convoluting the systems biology of the tick bite—that is determining the impact of virus, vertebrate host, and tick genetics (i.e., genome-by-genome-by-genome or GxGxG studies) on pathogenesis, is a priority. The diagnosis and treatment of tick-borne disease could be advanced by considering each tick bite as a “unique” molecular encounter between tick salivary proteins, the microbial flora delivered by the tick and host factors produced at the feeding wound (Figure 1). Molecular analyses support a human genetic component to the severity of TBF disease. Complete genome sequencing identified amino acid residues associated with severity of the Far-Eastern subtype (FE) strains of TBEV isolated from patients with encephalitic (Efd), febrile (Ffd), and subclinical (Sfd) forms of the disease (Belikov et al., 2014). Molecular studies revealed polymorphism in the salivary proteins secreted by individual unfed and feeding ticks (Wang et al., 2001), and specific combinations of vector and virus genotype were reported to affect vector competence (Lambrechts, 2011; Fansiri et al., 2013).

### Control of TBFs

**Figure 2:**
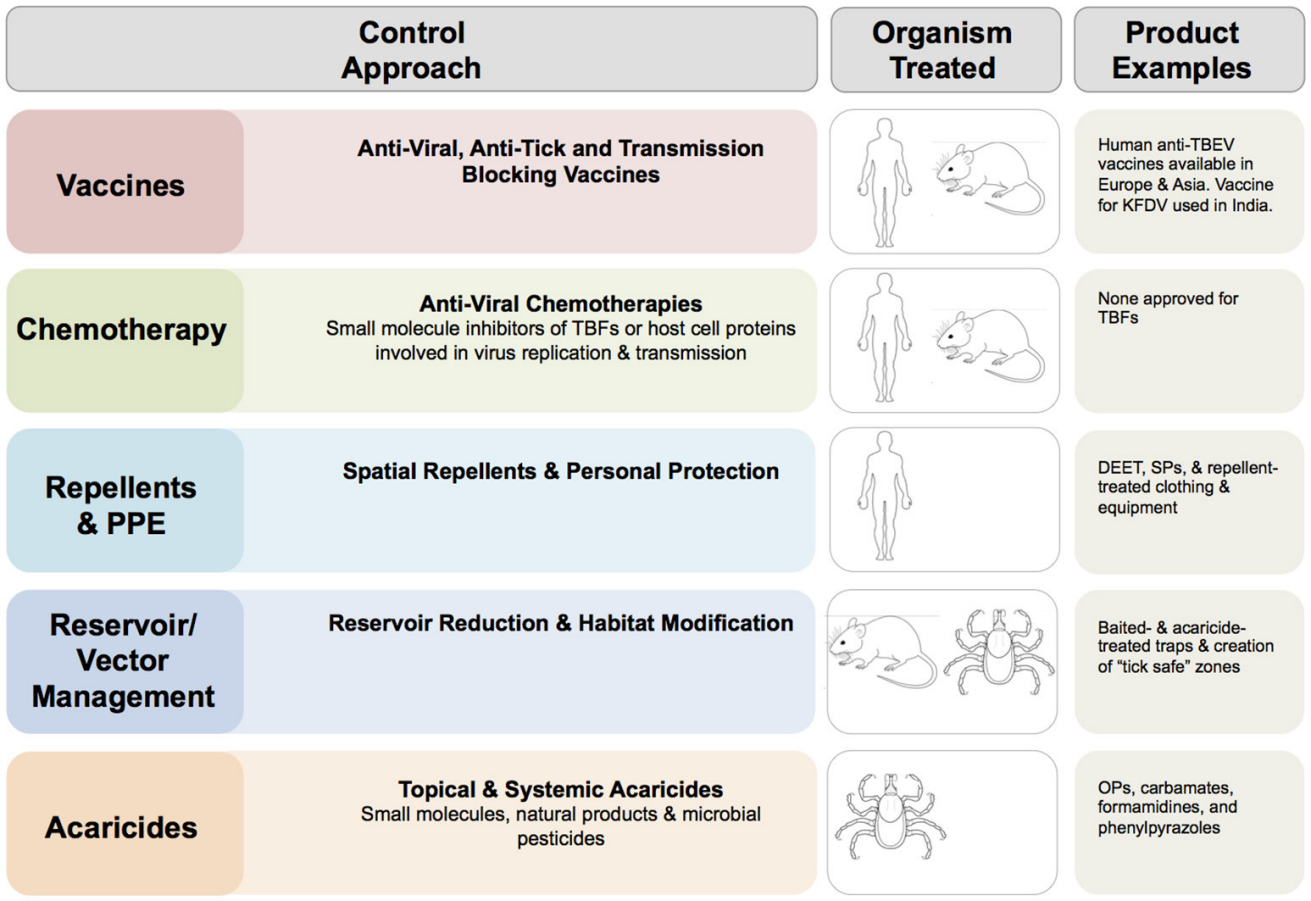
Options for control of tick-borne flaviviruses (TBFs). Common approaches for control of TBFs and examples of commercially available products are shown. DEET, N,N-Diethyl-meta-toluamide; OP, Organophosphate; POWV, Powassan virus; PPE, personal protective equipment; SP, Synthetic pyrethroid, TBEV, tick-borne encephalitis virus. The images of human, mouse and tick indicate the dead-end (human) host, non-human vertebrate reservoir and arthropod vector, as appropriate for virus in question.

Options to control TBFs (summarized in Figure 2) are limited and rely largely on personal protective measures, acaricides, vaccines against TBEV, and management of the symptoms of infection. Treatment of TBF infections in the human population focuses on palliative care and management of complications. There are currently no chemotherapies developed against TBFs. Viral infection may be treated with the antivirals (ribavirin, realdiron, larifan, and rifastin) developed to control a variety of human viral pathogens. Clinical studies to determine the effectiveness of these chemotherapies against TBF infection (Loginova et al., 2002; Lani et al., 2014) could have value.

Protective human vaccines are available for TBEV and KFDV. Currently, five products are considered safe and efficacious for protection against TBEVs. These are FSME-Immun and Encepur, manufactured in Austria and Germany respectively, and based on European strains of the virus, TBEV-Moscow, and EnceVir manufactured in the Russian Federation and based on FE strains (WHO), and the SenTaiBao vaccine manufactured in China (Xing et al., 2017) and also based on the FE subtype. These inactivated vaccines require multiple doses to induce and maintain immunity. The development of novel and more effective vaccines remains a high priority (Wang et al., 2016).

There is broad interest in transmission blocking vaccines for control of tick-borne diseases (reviewed below), although there are currently no products registered to prevent transmission of TBFs. The candidate 64TRP transmission blocking vaccine, based on a recombinant form of the 15 kDa cement protein of the African brown ear tick, *Rhipicephalus appendiculatus*, was associated with a reduction in TBEV transmission and disease in an *in vivo* mouse model (Labuda et al., 2006) and could have potential as a broad-spectrum anti-tick vaccine (Trimnell et al., 2005; Havlikova et al., 2009).

Acaricides are used to control ticks of public health and veterinary importance. Unfortunately, continued tick control is complicated by widespread resistance of tick populations to several classes of acaricides, most notably organophosphates (OPs) and carbamates (George, 2000; George et al., 2004; Abbas et al., 2014). The situation is most acute with respect to *R. microplus*. Large-scale application of chemicals has been effective (Ostfeld et al., 2006) for tick control in urban, rural and recreational areas but can also contribute to resistance and effects on vertebrates and other non-target species. Microbial insecticides based on the fungi *Metarhizium anisopliae* and *Beauveria bassiana* have been proposed as environmentally benign alternatives (Benjamin et al., 2002; Hornbostel et al., 2004, 2005; Ostfeld et al., 2006), and other “green” technologies are under consideration (Benelli et al., 2016). Insecticides based on plant-derived extracts are attracting attention as new classes of tick repellants and toxicants, and are the subject of ongoing mode of action studies (Gross et al., 2015, 2017).

Approaches to reduce transmission via management of either the vertebrate reservoir or the tick vector have been investigated. In the U.S., the topical application of acaricides delivered via baited applicators reduced densities of hard ticks on deer and small rodents in the field (Pound et al., 2000; Brei et al., 2009; Carroll et al., 2009; Miller et al., 2009). However, logistics and cost, including the need for constant maintenance of baited-field devices, suggest lack of feasibility at broader scale (Harmon et al., 2011). Passive acaricide applicators remain an option to reduce local tick burden when used in combination with other tick control strategies.

### Prospects for TBF-protective and anti-tick vaccines

Vaccines offer a cost-effective, sustainable, and environmentally friendly approach to control of arthropod-borne diseases, and in combination with drugs and insecticides, are the backbone of global disease control and eradication campaigns. Existing and developmental products suggest prospects for novel anti-TBF vaccines. Effective human vaccines for the prophylaxis of yellow fever (17D live attenuated virus), JEV (live attenuated and inactivated whole virus), and TBEV (inactivated whole virus; Heinz and Stiasny, 2012) are available. The live attenuated Dengvaxia® product developed by Sanofi Pasteur provides moderate protection against DENV1-4 strains and is approved for use in 11 countries. Efforts are also underway to develop vaccines against ZIKV (Abbink et al., 2016; Marston et al., 2016). Chimeric, recombinant, attenuated vaccines for TBEV have been investigated (Pletnev and Men, 1998; Pletnev et al., 2001; Wang et al., 2016). Live attenuated TBEV vaccines based on a replication-defective (single-cycle) flavivirus platform that provide efficacy after a single dose may be feasible (Rumyantsev et al., 2013). The development of recombinant, live vaccine candidates incorporating microRNA (miRNA) sequences may increase the effectiveness of live anti-TBF vaccines (Tsetsarkin et al., 2016, 2017). However, optimism for vaccine development is tempered by the theoretical risk of vaccine-related adverse events such as immune enhancement of infection and the requirement to induce a long-lasting protective immune response against multiple serotypes (Heinz and Stiasny, 2012).

In addition to novel anti-TBF vaccines, options may exist to “repurpose” existing vaccines and exploit cross reactivity for control of multiple virus species. Several studies suggest that TBEV vaccines may provide cross protection against other members of the TBF complex. There is evidence that immunization with the European/Western-based TBEV vaccine can reduce OHFV infection in mice and humans (Chidumayo et al., 2014), while immunization with the Russian-Spring-Summer Encephalitis virus (RSSEV) form of the TBEV vaccine was associated with a reduction in KFDV infection in mouse models (Aniker et al., 1962; Holbrook, 2012). Unfortunately, preliminary human vaccination studies with the RSSEV-based anti-TBEV vaccine suggested insufficient protection against KFDV (Pavri et al., 1962; Shah et al., 1962; Holbrook, 2012). Similarly, vaccination of mice with the TBEV-Moscow strain did not protect against POWV (Chernokhaeva et al., 2016; Doughty et al., 2017), suggesting limited potential for cross protection.

Anti-tick vaccines represent an effective and environmentally benign approach to control ticks and the pathogens they transmit (de la Fuente et al., 2016). The vaccines TickGARD and Gavac used to control *R. microplus*, a serious pest of cattle in the southern hemisphere and the vector of bovine babesiosis, are based on the Bm-86 midgut protein antigen of the tick. During tick feeding on an immunized host, the ingestion of host immunological factors is thought to induce lysis of tick midgut cells, thus reducing feeding and ultimately tick burden (Willadsen et al., 1989; Willadsen and Jongejan, 1999; Valle et al., 2004; Londono-Renteria et al., 2016). There is need to explore the potential of TickGARD and Gavac to reduce tick infestations on other vertebrate hosts. Vaccination against recombinant Bm-86 has been suggested as a strategy to reduce *R. microplus* infestations in white-tailed and red deer (Carreon et al., 2012), but there remain questions as to feasibility. TickGARD reduced transmission of TBEV from infected *I. ricinus* ticks to mice, but did not provide protection against infection (Labuda et al., 2006). TickGARD and/or Gavac may have efficacy against other vectors of TBFs, although cross-species activity has not been determined (Londono-Renteria et al., 2016). The 64TRP candidate may have potential as a vaccine to prevent TBEV transmission; mice immunized with the recombinant protein and exposed to infected *I. ricinus* were protected against lethal challenge with TBEV (Labuda et al., 2006) but the potential scope of protective immunity provided by the vaccine (i.e., the tick species and TBFs controlled) requires investigation.

Strategies for *de novo* development of anti-tick vaccines are under investigation. Vaccines against concealed or exposed tick antigens could reduce TBF pathogen load in vector and reservoir, host exposure to ticks, and tick populations (Nuttall et al., 2006; de la Fuente and Merino, 2013). Theoretically these products could be delivered via the vertebrate host (e.g., via oral bait to wildlife or by vaccination of humans and domestic animals). Proteins associated with feeding, reproduction, development, immune response, subversion of host immunity, and that are vital for pathogen infection and transmission have been suggested as candidate protective antigens (Contreras et al., 2016). Multiple recombinant tick proteins are under investigation as candidates for vaccines to control ticks and are described in recent reviews (de la Fuente and Contreras, 2015; de la Fuente et al., 2016). The aquaporin trans-membrane proteins involved in transport of solutes and water, ferritin 2 (Fer2) iron regulating proteins and 64TRP are considered some of the most “promising” candidate antigens (Hussein et al., 2015; de la Fuente et al., 2016). These proteins are associated with a variety of physiological functions; in challenge studies they provided protection against multiple species of ticks, and the potential for immunogenic protection against pathogen transmission is now under investigation. Combinatorial products have also been proposed that would deliver multiple antigens to control transmission of several pathogens (de la Fuente et al., 2016).

High-throughput vaccine discovery platforms have been proposed. Transcriptomic and proteomic data provide a starting point for identification of candidate protective antigens from *I. scapularis* (Contreras et al., 2016). Bioinformatics-based reverse vaccinology approaches (i.e., *in silico* predictions of antigenic epitopes based on *ab initio* gene models or “omics” datasets) are described in a recent review (Lew-Tabor and Rodriguez Valle, 2016).

### Prospects for TBF antivirals

Some progress has been made toward the development of antivirals (Patkar and Kuhn, 2006). Nucleoside analogs have been studied for control of arthropod-borne flaviviruses (Yin et al., 2009) and could help to expand the toolbox of small molecule inhibitors of TBEV. Structure-activity relationship (SAR) studies have identified nucleoside moieties that may inhibit entry of the virus to the host cell or interaction with the non-structural protein 5 methyltransferase and the RNA-dependent RNA polymerase domains of TBEV (Orlov et al., 2017). Drug “repurposing” could expand chemical control options for TBFs. The NITD008 adenosine analog active against mosquito-borne flaviviruses and POWV (Yin et al., 2009) exhibited antiviral activity against KFDV, AHFV, and OHFV *in vitro*, while the BCX4430 analog suppressed WNV, TBEV, LIV, and KFDV *in vitro*, suggesting the potential to suppress “pan-flaviviral activity” (Lo et al., 2016; Eyer et al., 2017).

Small molecule chemistries that target the envelope proteins (E proteins) of TBFs have potential as antivirals (Zhou et al., 2008; Mayhoub et al., 2011a,b). E proteins are involved in virus infection of the host cell, and virus assembly and morphogenesis. The crystal structure of the soluble ectodomain of the DENV type 2 E protein revealed a hydrophobic pocket lined by residues that influence the pH threshold for virus fusion with host cells. Features of the pocket point to a structural pathway for the fusion-activating transition and a mechanism that could be targeted by small-molecule inhibitors of flaviviruses (Modis et al., 2003). The phenylthiazole ring system has emerged as a template for design of antivirals. Virtual screening of the National Cancer Institute (NCI) drug database combined with medicinal chemistry strategies identified small molecules that may be active at this target (Li et al., 2008). Analogues that preserve antiviral activity while reducing adverse effects could provide a new class of antivirals against TBFs.

### Prospects for novel acaricides

Insecticides are effective tools for control of vector-borne diseases. Unfortunately, widespread resistance among pest populations represents a threat to continued disease control. The identification of pesticide chemistries that operate via novel modes of action (MoA) by binding at alternative sites on existing insecticide targets) or via disruption of novel molecular targets in the arthropod, is a high priority (Van Zee and Hill, 2017). Disease control is expected to rely on insecticides for the next several decades and new acaricides that operate via targets distinct from acetylcholinesterase (the main target of OPs and carbamates) and the voltage-gated sodium channel (the main target of SPs) are sought. The Innovative Vector Control Consortium (IVCC) has issued a call for three new MoA insecticides by 2023 to control mosquito vectors of malaria (Hemingway et al., 2006). We suggest that a similar challenge would also be appropriate for control of ticks and TBFs.

The availability of genome data permits target-based approaches to acaricide discovery. For example, the “genome-to-lead” approach (Meyer et al., 2012) was employed to identify small molecule antagonists of an *I. scapularis* dopamine receptor (DAR). The target was selected from several hundred G protein-coupled receptors (GPCRs) predicted from the IscaW1.1 assembly (Gulia-Nuss et al., 2016). High throughout chemical screening (HTS), followed by “hit-to-lead” and structure-activity studies (SAR) were used to discover several chemistries with high *in vitro* potency for the receptor (Meyer et al., 2011; Ejendal et al., 2012) that may provide leads for new pesticides. Research has also focused on pharmacological characterization of the *R. microplus* octopamine receptor, a suspected target of botanical insecticides (Gross et al., 2015, 2017), and an *I. scapularis* ligand-gated chloride channel considered the target of ivermectin (Gulia-Nuss et al., 2016). These proteins could be used in small molecule screens and targeted by genetic control strategies based on dsRNA/siRNA-mediated RNAi knock-down or Crispr/Cas9 knock out, although protocols for efficient tick transformation would be required for success of the latter.

### Tick-virus “interactomics”; understanding pathogenesis, and identifying new vaccine and acaricide targets

The identification of protein targets is a major roadblock to development of novel anti-TBF and transmission blocking vaccines and acaricides; it is here that “omics” research may have greatest impact. Genomics has aided understanding of tick-pathogen interactions and rapid identification of multiple candidate protein targets *en-masse*. Systems biology studies have identified metabolic pathways and enzymes perturbed during viral infection of cells. These studies could help to pinpoint proteins critical to cellular invasion, replication, and transmission of the virus. Transcriptomic and proteomic analyses have focused on the mosquito-DENV (Behura et al., 2011; Bonizzoni et al., 2012; Chauhan et al., 2012; Chisenhall et al., 2014) and tick-*Anaplasma phagocytophilum* (bacterium that causes human granulocytic anaplasmosis) (Ayllon et al., 2015b; Villar et al., 2015; Alberdi et al., 2016; Cabezas-Cruz et al., 2017) “interactomes”. The limited concordance between these studies highlights the value of equivalent research in tick-virus systems.

**Figure 3:**
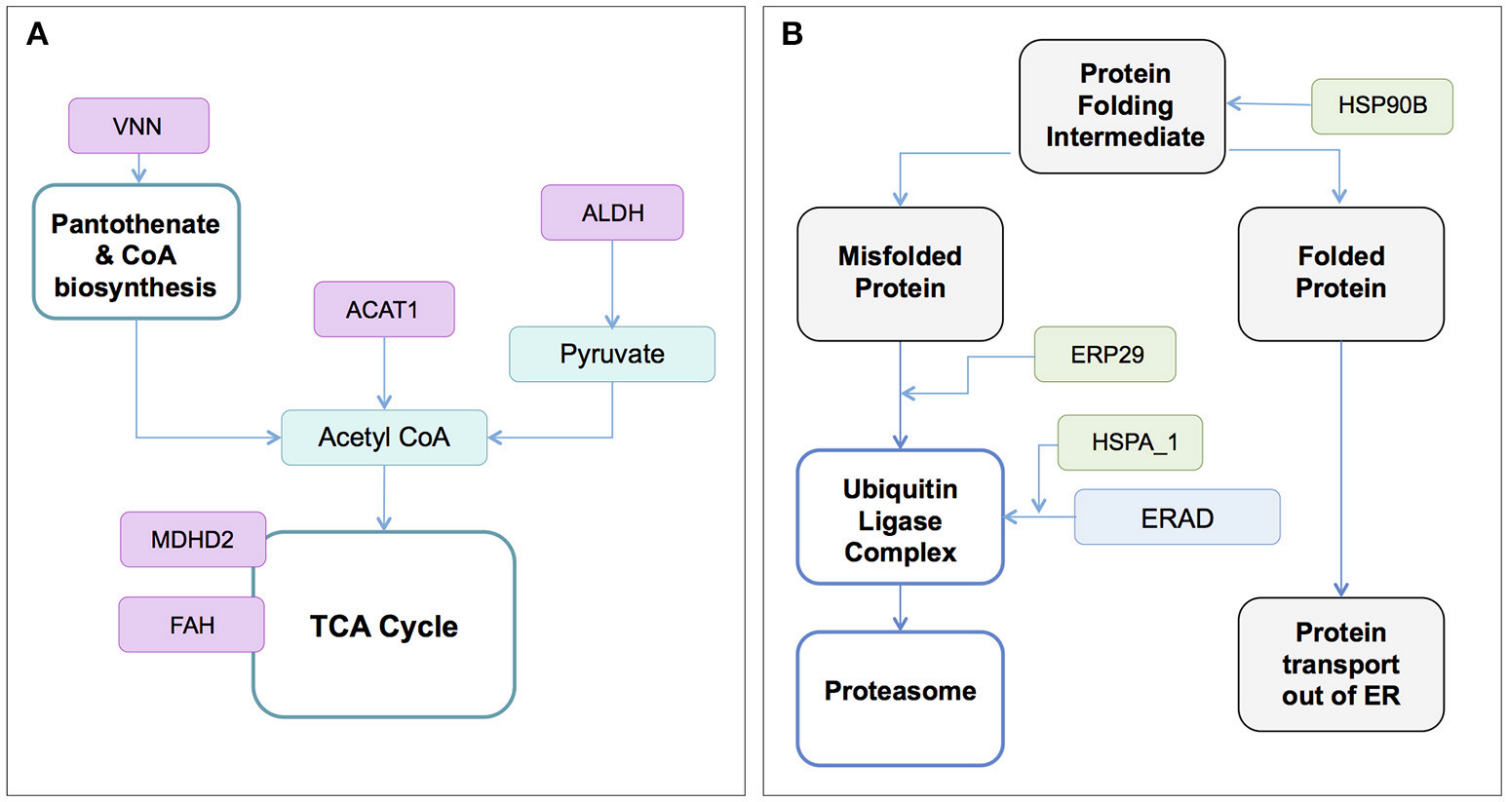
Enzymes and biochemical/metabolic pathways associated with the infection and replication of the tick-borne flavivirus, LGTV (Weisheit et al., 2015; Grabowski et al., 2017a). RNAi-induced knockdown of transcripts for proteins identified to **(A)** the pantothenate and CoA biosynthesis, and TCA cycles, and **(B)** Protein folding and degradation processes was associated with reduced LGTV infection in *Ixodes scapularis* ISE6 cells. Viral infection was assessed by the end points of viral genome replication and infectious virus release. Biosynthetic pathways (teal or blue rectangles), protein states (gray shaded rectangles) and enzymes/proteins (magenta or green rectangles) are shown. VNN and ACAT1 reduced LGTV genome replication and viral replication, while ALDH, MDH2, and FAH reduced LGTV replication only. HSP90B (ISCW022766); ERP29 (ISCW18425); HSP1_8 (ISCW024057, ISCW024910); VNN, (ISCW004822); ACAT1 (ISCW016117); ALDH (ISCW015982); MDH2 (ISCW003528); FAH (ISCW020196). ACAT, acetyl-CoA acetyltransferase; ALDH, aldehyde dehydrogenase; ER, endoplasmic reticulum; ERAD, endoplasmic reticulum-associated degradation; ERP29, endoplasmic reticulum protein 29; FAH, fumarylacetoacetate hydrolase; HSPA1_8, heat shock protein 70 family A members 1-8; HSP90B, heat shock protein 90 beta family; MDH2, malate dehydrogenase 2; TCA, tricarboxylic acid.

Several transcriptome and proteome studies have analyzed the global response of tick cell lines to infection with TBFs and identified protein candidates for vaccine and acaricide development (Weisheit et al., 2015; Grabowski et al., 2016, 2017a). These studies were conducted using TBEV or the less pathogenic Langat virus (LGTV; Table 1; McNally et al., 2012; Weisheit et al., 2015; Grabowski et al., 2016). The involvement of multiple biochemical pathways was suggested following viral infection, with perturbation of pathways for protein folding and degradation, and metabolic processes (Figure 3). Some of these pathways have also been implicated in studies of mammalian cells exposed to HCV, DENV, and JEV (Table S1), suggesting the involvement of common cellular responses to flavivirus infection and potential for vaccines with cross-protective immunity. Metabolic pathways have also been investigated in studies of other host-flavivirus (Diamond et al., 2010; Perera et al., 2012; Fischer et al., 2013; Merino-Ramos et al., 2015) and tick-pathogen (Cabezas-Cruz et al., 2017) systems. Proteomic and metabolomics studies also have the potential to uncover proteins and pathways that are unique to the infectious state, an area of research that deserves further attention.

RNAi was used to investigate the role of tick proteins in LGTV infection of the ISE6 cell line derived from *I. scapularis* (Grabowski et al., 2017a) (Figure 3, Figure S1). Results suggest involvement of proteins that mapped via *in silico* methods to pathways for amino acid, carbohydrate, lipid, cofactor and vitamin, terpenoid, and polykeytide metabolism. Proteins associated with processing in the endoplasmic reticulum (ER) may also function to facilitate or suppress virus infection (Figure 3, Figure S2; Weisheit et al., 2015; Grabowski et al., 2017a). Future work must distinguish metabolic changes associated with direct manipulation of the host cell by the virus versus the generalized cellular stress response. The involvement of orphan proteins reported in LGTV infected *I. scapularis* ISE6 cells (Grabowski et al., 2017a), highlights the need to characterize “hypothetical” proteins predicted by “omic” studies.

One priority is to understand the biology of TBFs in the context of the tick tissues and cells associated with primary and secondary cycles of virus infection and replication. *In vivo* studies have validated several protein targets in tick tissues and whole ticks (Narasimhan et al., 2004; de la Fuente et al., 2005; Karim et al., 2010; Kocan et al., 2011). Electron tomography studies have investigated the three-dimensional architecture of structures derived from host cell membranes that form during DENV infection and replication in mosquito and human cells (Junjhon et al., 2014), and multiple studies suggest virus manipulation of host lipid pathways and cellular membranes (Heaton et al., 2010; Perera et al., 2012; Jordan and Randall, 2016). Similar studies have been performed in tick cells exposed to TBFs (Senigl et al., 2006; Offerdahl et al., 2012; Hirano et al., 2014; Bily et al., 2015) and investigations focused on tissues such as the midgut and salivary glands are needed. TBF infection and spread has been demonstrated in short-term culture of *I. scapularis* organs, providing a platform for tissue-specific studies of virus infection (Grabowski et al., 2017b). Phosphorylation and acetylation of host proteins has been associated with viral infection (Liu et al., 2014; Jeng et al., 2015; Oberstein et al., 2015; Ohman et al., 2015). Metabolomic studies are expected to improve understanding of how post-translational modification (PTM) of host proteins affects viral replication and transmission, consider another area of research priority.

Review of tick-virus “interactome” studies reveals several gaps and impediments to the research goals outlined in this manuscript. Firstly, “omic” research must expand beyond the *Ixodes*-TBF model to other tick-TBF systems (see Table 1), emphasizing major vectors and high consequence pathogens. Unfortunately, the biosafety level of the more pathogenic TBFs such as TBEV and POWV restricts research to institutions with appropriate containment facilities. To ensure that data are relevant in a biological context, the field must develop community resources and *in vivo, ex vivo*, and *in vitro* research tools reflective of the vector species and viruses involved. Multiple tick cell lines derived from vectors of TBFs are available for *in vitro* studies via the Tick Cell Biobank (Bell-Sakyi et al., 2007) and in-bred laboratory colonies of ticks competent for TBF transmission must be established. To provide frameworks for resource development, the role of tick species in virus transmission must be addressed via natural history and vector incrimination studies (Nuttall and Labuda, 2003).

### The next frontier: structural genomics and paradigm shifts in HTP vaccine, drug, and acaricide discovery platforms

The selection of suitable antigens is a major constraint to vaccine development (Havlikova et al., 2009). Target-based antiviral and acaricide discovery also requires validated protein targets amenable to high-throughput (HTP) virtual (i.e., *in silico*) or compound library screening. Advances in structural genomics could facilitate radical changes in HTP discovery platforms for new technologies to control TBFs. Structural genomics is enabling experimental characterization of the three dimensional (3-D) atomic structure of proteins and other molecules having an important biological role in human infectious diseases. Experimental 3-D protein structures and protein-ligand complexes have been generated for organisms causing emerging and re-emerging diseases, including CDC Category A-C priority agents, by the techniques of X-ray, nuclear magnetic resonance (NMR), and cryo-electron microscopy (cryo-EM). These technologies have enabled molecular screening of proteins in complex with inhibitors, cofactors, and substrate analogs, with data from structural studies used to guide virtual screening (Wang et al., 2015).

The past decade has seen an explosion of studies to determine the structure of arthropod-borne flaviviruses in both mature and immature state (Table 2). Knowledge of virion structure, assembly and cellular entry mechanisms can support prediction of antigenic epitopes for rational design of vaccines and *in silico*, structure-based discovery of drugs that interfere with viral entry and replication (Patkar and Kuhn, 2006). Cryo-EM has enabled resolution of virion architecture for DENV, WNV, and ZIKV (Heinz and Stiasny, 2012) (Table 2). Structural investigations involving TBFs are limited to X-ray crystallography and NMR studies of the TBEV and LGTV E glycoproteins (Rey et al., 1995; Mukherjee et al., 2006), considered the most important immunogen. Homology modeling and molecular docking have been used to identify inhibitors of TBF reproduction, with several compounds showing inhibition of POWV and TBEV *in vitro* (Osolodkin et al., 2013). The structure of human antibodies in complex with ZIKV (Hasan et al., 2017) and DENV (Pokidysheva et al., 2006; Lok et al., 2008) has been determined, suggesting potential for development of neutralizing antibodies. Greater knowledge of virion structure will enable equivalent studies for TBFs.

**Table 2:** Summary of structural studies of Flaviviridae transmitted by arthropods.

**Virus and Strain**	**Year**	**Resolution (Å)**	**Reference(s)**	**Protein Data Bank (PDB) Accession(s)**
**DENGUE VIRUS (DENV)**				
DENV2 S1 strain^1^	2002	24	Kuhn et al., 2002	1K4R
DENV2	2003	9.5	Zhang et al., 2003	1JCH/1P58/1SVB
DENV2	2013	3.5	Zhang et al., 2013	3J27
DENV1	2013	4.5	Kostyuchenko et al., 2013	4B03/4AZX
DENV4	2014	4.1	Kostyuchenko et al., 2014	4CBF
**WEST NILE VIRUS (WNV)**				
NA^2^	2007		Zhang et al., 2007	2OF6
NY 1999^1^	2003		Mukhopadhyay et al., 2003	–
**ZIKA VIRUS**				
H/PF/2013^1^	2016	3.8	Sirohi et al., 2016	5IRE
NA^2^	2017	9	Prasad et al., 2017	5U4W

*Crystal structure of virus E glycoprotein available; DENV (Modis et al., 2003), JEV (Luca et al., 2012), LGTV (Mukherjee et al., 2006), TBEV (Rey et al., 1995) and ZIKV (Prasad et al., 2017) and virus structure in complex with other proteins (Zhang et al., 2015)*.

1 *Denotes structure of mature virus*.

2 *Denotes structure of immature virus*.

**Figure 4:**
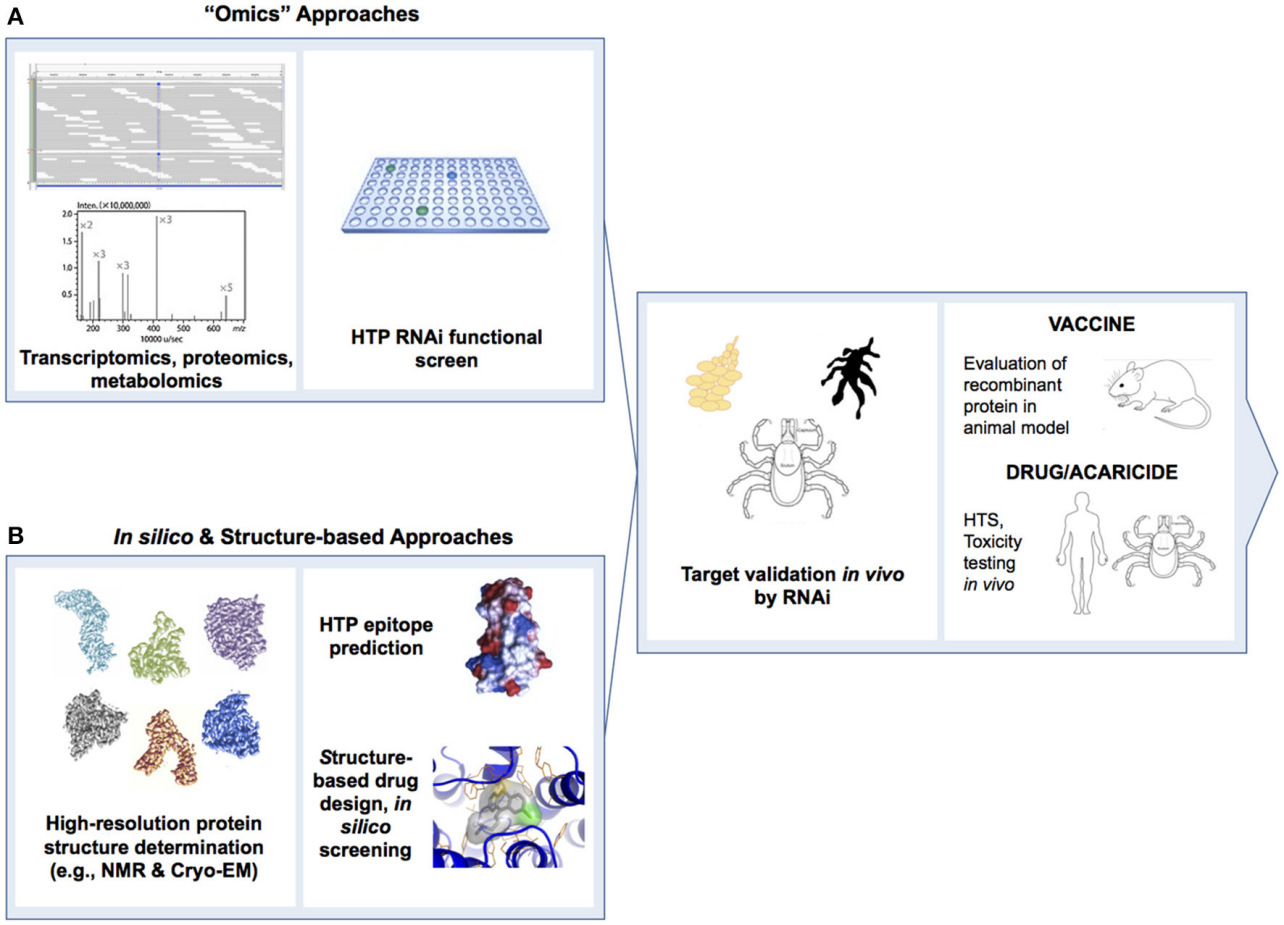
Schematic depicting the major steps in **(A)** wet-lab “omic” and **(B)** *in silico*-processes to identify tick protein targets for development of transmission blocking vaccines, antivirals and acaricides to control TBFs. Antigenic virus or tick proteins identified in **(A,B)** would proceed to vaccine clinical trial. Virus and tick proteins identified in **(A)** would proceed to high-throughput screen (HTS) development and identification of small molecule drugs and acaricides. Tick proteins identified in **(B)** would proceed to pharmacological assays and development of additional HTS. Third panel from left depicts RNAi functional studies in tick salivary glands and midgut, and whole ticks. Cryo-EM, cryo-electron microscopy; HTP, high-throughput; HTS, high-throughput screen; NMR, nuclear magnetic resonance; RNAi, RNA interference.

Structural genomic studies of tick proteins could generate data for the rational design of next generation transmission blocking vaccines and acaricides (Figures 4, 6). Crystal structures are available for a salivary cystatin from the soft tick, *Ornithodorus moubata* (Salat et al., 2010) and a thrombin from the Tropical Bont tick in complex with S-variegin (Koh et al., 2011). The development of HTP platforms for protein expression and purification could permit atomic level resolution of structures for soluble tick proteins. Advances in techniques for the genetic manipulation of arthropods such as the Crispr/Cas9 gene editing technology could facilitate HTP validation of protein targets *in vivo*. Paradigm shifts in the approach to vaccine and acaricide discovery are expected. Future efforts are likely to incorporate (1) systems biology studies to identify novel protein targets *en masse*, (2) *in vitro* validation of multiple protein targets in parallel via RNAi or Crispr/Cas9 screens, (3) structure-based and virtual screening, and (4) *in vivo* functional studies in tick tissues and whole ticks (see Figure 4).

### Forward genetics to understand tick vector competence and identify genetic elements associated with TBF transmission

Forward genetics (i.e., “phenotype to gene studies”) represents a powerful approach to identify loci associated with phenotypes such as acaricide resistance, tick host preference and vector competence (Meyer and Hill, 2014). Reverse genetics (i.e., the “gene to phenotype studies” described above) has advanced understanding of the function of tick gene products, yet the “major players”—those gene products critical to viral infection, replication, and transmission, remain elusive. The feasibility and cost of developing genetic resources has stymied forward genetics of ticks. Below, we discuss the potential of forward genetics for tick-virus research, and the resources required to support this work.

Genetic mapping and genome wide association studies (GWAS) are techniques employed to identify quantitative trait loci (QTL) associated with key phenotypes. Genetic studies have been used to investigate mosquito-virus systems. Genetic differences among populations of the *Aedes aegypti* mosquito vector of DENV, ZIKV, yellow fever, and CHIKV were correlated with vector competence for flavivirus transmission (Black et al., 2002). For example, QTL for the “midgut infection barrier” phenotype associated with reduced DENV2 serovar infection of *Ae. aegypti* were mapped to several chromosomes and found to account for a significant percentage of the phenotype (Bosio et al., 2000; Gomez-Machorro et al., 2004). Fine-scale mapping, map-based positional cloning and functional studies are typical next steps to identify genes associated with QTL.

**Figure 5:**
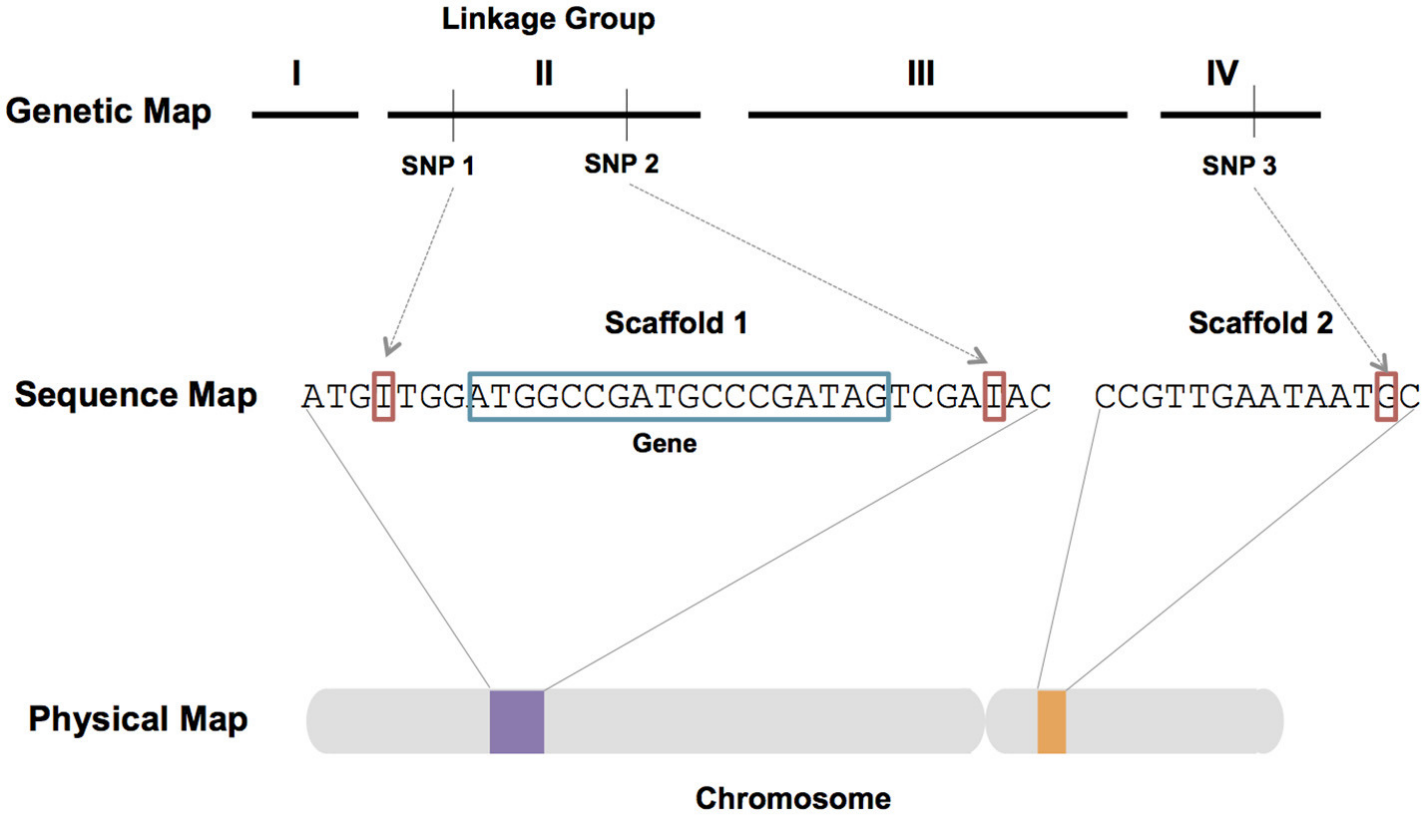
Schematic diagram showing the integration of genetic, sequence and physical maps. Genetic markers such as single nucleotide polymorphism (SNP) markers enable the association of assembled sequence reads with genetic linkage groups. Sequence can be oriented on chromosomes via physical mapping. Integrated maps and fine scale genetic mapping techniques can be used to identify regions of the genome associated with quantitative trait loci (QTL) and genes associated with phenotypes of interest.

Assembled genomic sequence coupled with expression data, genetic (linkage) maps, and physical maps (Figure 5) represent key resources for genomic research. Currently, the *I. scapularis* IscaW1 assembly (ABJB010000000) is the only genome assembly for a tick that comprises sequence scaffolds of Mb length. The assembly consists of 369,495 scaffolds that provide ~ 3.8X coverage of the 2.1 Gbp haploid genome. Annotation of scaffolds representing ~57% of the genome, revealed 20,486 protein-coding genes and expansions of gene families associated with tick–host interactions. Improvement of the *I. scapularis* assembly and the generation of draft assemblies for other tick species are high priorities. However, haploid genome size and complexity make this a costly and challenging goal (Meyer and Hill, 2014). The haploid genomes of multiple hard and soft tick species are estimated to exceed 1 Gb, and typically comprise relatively high levels of repetitive DNA sequence as compared to many arthropods (Ullmann et al., 2003; Geraci et al., 2007; Meyer and Hill, 2014; Gulia-Nuss et al., 2016). Third generation genomic technologies such as long-read sequencing (PacBio and Hi-C) and optical mapping (Jiao and Schneeberger, 2017) are expected to enable chromosome-level assemblies for ticks. Optical mapping is ideally suited for the improvement of fragmented genome assemblies and scaffolding of *de novo* assemblies from high throughput sequence reads (Howe and Wood, 2015). These technologies have been used to generate an improved assembly for *Ae. aegypti* (Dudchenko et al., 2017) and will likely be useful to generate genome assemblies for tick species.

Future genome sequencing targets identified by the Tick and Mite Genomes Consortium are described in a white paper (Hill, 2010; Van Zee and Hill, 2017). This project, approved by the National Institutes of Health, is a community-ratified guide for genomic and genetic research on ticks and mites of medical and veterinary importance. The whitepaper proposes sequencing of species representing the major lineages comprising the subclass Acari (ticks and mites). Beachhead species include (1) the prostriate vectors of TBFs in Europe and Asia, *I. ricinus* and *I. persulcatus*, (2) the metastriate ticks *Dermacentor variabilis* (American dog tick), the vector of the *Rickettsia rickettsia* bacterium that causes Rocky Mountain Spotted Fever (RMSF) and *A. americanum* (lone star tick), the vector of erlichiosis and *Borrelia* spp, (3) the soft tick *Ornithodoros moubata* (family Argasidae), and (4) the *Leptotrombidium deliense* mite vector of scrub typhus (Superorder Acariformes). These species represent key phylogenetic nodes, and were selected based on their significance as vectors and potential to nucleate additional genomic research.

Forward genetics requires mapping populations (i.e., in-bred laboratory lines with quantifiable traits), large numbers of molecular markers for coarse and fine-scale mapping, and high-density genetic maps. The development of mapping populations of ticks has been stymied by the relatively long lifecycle of many species and the costs associated with colony maintenance. Multiple types of molecular markers have been produced for species of tick vectors (Meyer and Hill, 2014; Araya-Anchetta et al., 2015). Notably, thousands of single nucleotide polymorphism (SNP) markers have been identified from populations of *I. scapularis* using the technique of *R*estriction Site-*A*ssociated *D*NA sequencing (RADseq) (Gulia-Nuss et al., 2016) and PCR (Van Zee et al., 2013, 2015). The preliminary *I. scapularis* linkage map, generated according to the segregation of 127 loci in 232 F1 intercross progeny from a single female tick and using a combination of RAPD, sequence-tagged RAPD (STAR), cDNA, and microsatellite markers, represents the only such resource for any tick (Ullmann et al., 2003). Fourteen linkage groups were identified that may correspond to the haploid number of chromosomes in *I. scapularis*. The map of 606 centimorgans (cM) had a marker interval of 10.8 cM and the estimated relationship of physical to genetic distance was ~663 kb/cM. More than 7 M SNPs identified via the study of Gulia-Nuss et al. (2016) and available at VectorBase (www.vectorbase.org/) provide a basis for development of a high-density linkage map for *I. scapularis*. Such maps should also be the goal for other TBF vectors.

Physical mapping is a complementary technique to assign and orient sequence data on chromosomes, integrate sequence and genetic maps, and improve genome assemblies (Figure 5). Physical maps support cytogenetic research, including the development of karyotypes and studies of chromosome synteny. Chromosome number has been determined for multiple species of ticks (Oliver, 1977) providing insights into reproductive strategies among members of the Acari. Physical mapping using species of repetitive DNA and assembled sequence data have enabled investigations of genome organization for pro- and metastriate ticks (Meyer and Hill, 2014). Preliminary physical maps were produced for *I. scapularis* and *R. microplus* using the technique of fluorescent *in situ* hybridization (FISH) to study the chromosomal arrangement of families of tandem sequence repeats (Hill et al., 2009; Meyer et al., 2010; Gulia-Nuss et al., 2016). Physical maps must be developed for additional species of hard and soft ticks to support genome research on a range of TBF vectors.

An understanding of population structure and dynamics is critical for determining the role of ticks in disease transmission and for modeling and managing new control strategies. Studies of genetic diversity have been reported for at least 22 tick species representing six genera and the families Argasidae and Ixodidae. In the last several decades, the development of molecular markers has permitted the resolution of phylogenetic relationships at different taxonomic levels and population genetic analyses for multiple species (reviewed in Araya-Anchetta et al., 2015). Observed levels of population genetic structure range from negligible to high across the Ixodida, and for some species, suggest a correlation to host movement and significant host-race adaptation. Increasingly, research is directed at the contribution of tick population structure to the diversity and phylogeography of the pathogens they transmit, and the implications for disease risk (Qiu et al., 2002; Girard et al., 2009; Humphrey et al., 2010; Swei et al., 2015). Collectively, genetic mapping, GWAS and population genomic studies should enable the identification of loci that contribute to TBF transmission.

### Priority areas for research investment

**Figure 6:**
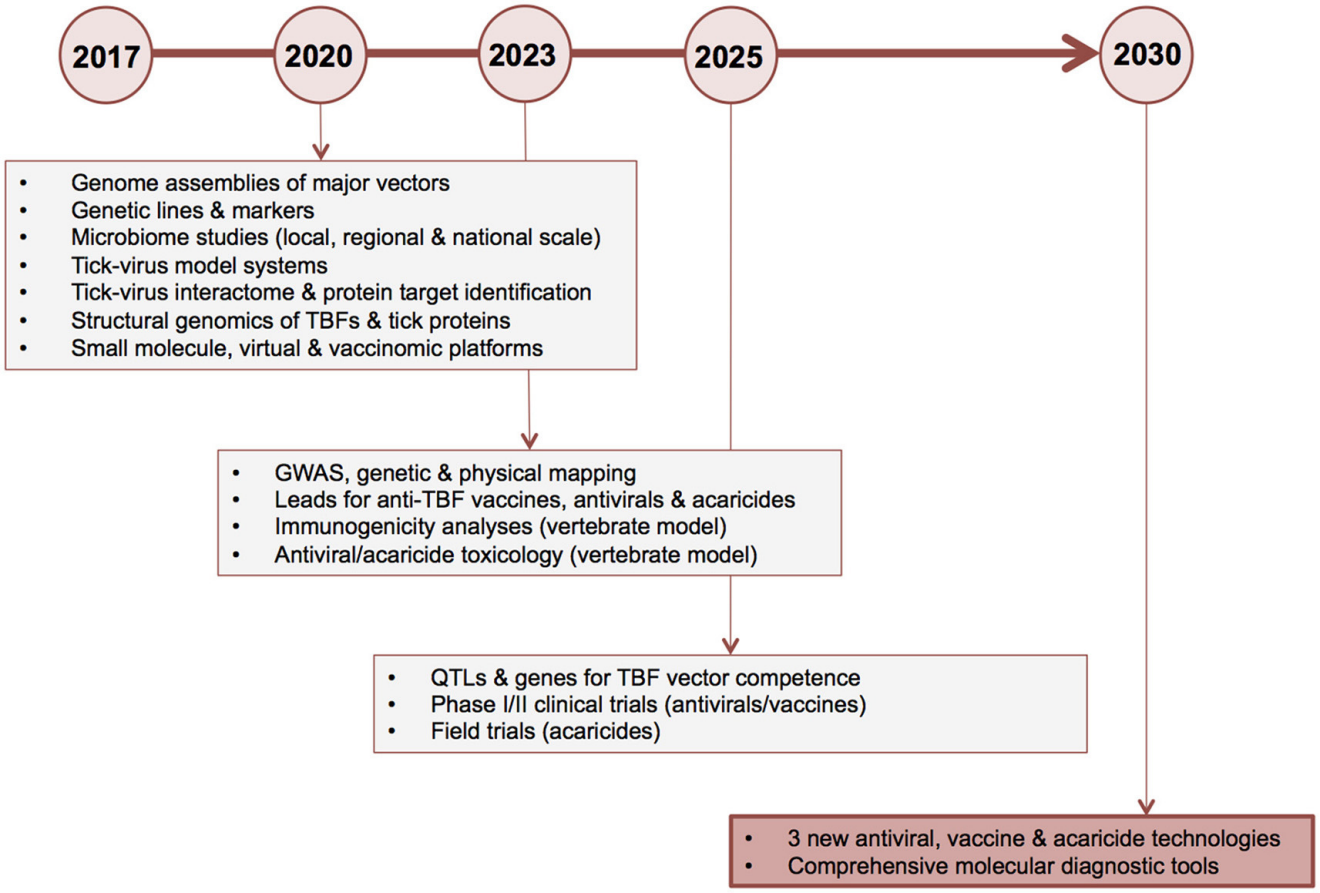
Ten-year roadmap for “omics” research to combat tick-borne flaviviruses. The proposed timeline for delivery of new antiviral, vaccine and acaricide control technologies by a target date of 2030 is shown on the horizontal axis. Key deliverables (boxed text) and corresponding major milestone dates of 2020, 2023, 2025, and 2030 (circles) are shown. GWAS, genome-wide association studies; HTP, high-throughput; HTS, high throughput screening; TBF, tick-borne flavivirus; QTL, quantitative trait loci.

Below, we suggest priorities for “omics” research and outline a proposed roadmap for delivery of new TBF control technologies by a target date of 2030. We challenge the field to develop three or more vaccine candidates and three or more leads for novel antivirals and acaricides within this timeframe. Key deliverables and proposed milestone dates are shown in Figure 6.

#### Research on TBFs

1. Metagenomic studies to define the complement of viral phyla and the prevalence of TBFs in the microbiome of tick vectors at regional scale.
2. Studies to determine the pathogenicity of viruses circulating in tick populations.
3. Studies to understand the systems biology of individual tick bites and the molecular interplay between microbial complement and tick and vertebrate host factors (i.e., GxGxG studies; Figure 1).
4. Precision medicine and improved passive surveillance for TBFs; the development of comprehensive molecular diagnostic tools (i.e., wearable devices, point of care diagnostics, and field sensors that detect hundreds rather than tens of pathogens, coupled with disease risk matrices to guide health care delivery).
5. Cryo-EM structural studies of high consequence TBFs and virus-antibody complexes.

#### Research on tick vectors of TBFs

1. Field studies focused on elucidation of natural TBF transmission cycles and incrimination of tick vector and reservoir species.
2. Prioritization of research in biologically relevant “tick-virus” systems; the development of resources including in-bred tick strains and tick cell lines derived from major tick vectors, and pathogenic viral species and strains. Dissemination of resources to the scientific community via resource sharing platforms such as the NIH funded BEI Resources (https://www.niaid.nih.gov/research/bei-resources-repository).
3. Development of resources for genome research on high priority tick vectors, including:
  - Improvement of the exitsing *I. scapularis* IscaW1 reference genome assembly (Gulia-Nuss et al., 2016) using third-gen technologies.
  - Production of high quality draft genome assemblies for “node” species, including representatives of the pro- and metastriate lineages, the major genera of hard (*Ixodes, Dermacentor, Amblyomma, Hyalomma, Rhipicephalus*) and soft (*Ornithodorus*) ticks, and the major vectors of tick-borne diseases in Europe, Asia, and the Americas (see Table 1 and Hill, 2010).
  - Generation of “omic” (transcriptomic, proteomic, and metabolomic) datasets for major tick vectors to support gene annotation, protein prediction and pathway analyses.
4. Structural genomic studies of the tick proteome via cryo-EM and crystal structures of key tick proteins to support *in silico* research.
5. Development of resources to support tick genetics and population genomics research, including:
  - Mapping populations of tick species with quantifiable traits, with an emphasis on strains that exhibit differences in vector competence and capacity for transmission of TBFs.
  - Genetic markers (e.g., SNPs) for genetic mapping, GWAS, population genomics and phylogenomics.
  - High density genetic and physical maps for major vector species (Figure 5).

#### Research on vaccines, antivirals, and acaricides to control TBFs

1. Radical redesign of discovery pipelines incorporating virus and tick protein targets and rational, *in silico* design of vaccines, antivirals and acaricides (see Figure 4).

### Conclusions: potential at the convergence of forward and reverse genetics

Genome assemblies provide an essential framework to support both forward and reverse genetics on ticks. In coming years, the field will witness additional tick genome projects, including assemblies for tick vectors of TBFs. Omic studies must emphasize tick-virus systems and will expand to include metabolomics. Structural studies embracing tick and TBF proteins will enable the redesign of drug discovery pipelines. Finally, it is hoped that forward tick genetics will become a reality, and converge with reverse genetic strategies to permit identification of the gene products associated with transmission of TBFs. Thus positioned, the field can realistically expect a paradigm shift toward precision medicine, and realization of the overarching objective long promised by genomics—the improved control of TBFs.

## Author contributions

JG and CH conceived the study, wrote the paper and approved the manuscript.

### Conflict of interest statement

The authors declare that the research was conducted in the absence of any commercial or financial relationships that could be construed as a potential conflict of interest.
