# Supplementary material for: A Roadmap for Tick-Borne Flavivirus Research in the “Omics” Era
Source: Front Cell Infect Microbiol. 2017 Dec 22;7:519. doi: 10.3389/fcimb.2017.00519 (PMC5744076; doi:10.3389/fcimb.2017.00519)
Supplement: Supplementary file 1 [file DataSheet1.DOCX]

**Supplementary Material**


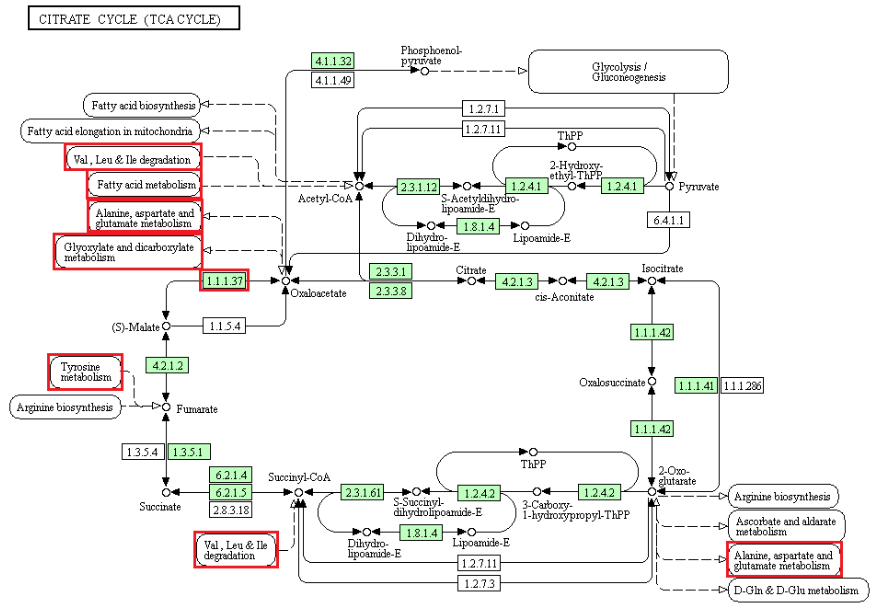


**Figure S1. Enzymes mapped to TCA cycle and metabolic pathways associated with infection of tick cells by the flavivirus, LGTV.** Boxes denote genes assigned via Kyoto Encyclopedia of Genes and Genomes (KEGG) software ([Kanehisa et al., 2017](#_ENREF_83)) to “citrate cycle (TCA cycle)” and pathways associated with the TCA cycle. Green boxes denote orthologous gene models identified from BLAST similarity search of the *Ixodes scapularis* IscaW1 gene model set ([Gulia-Nuss et al., 2016](#_ENREF_61)) downloaded from VectorBase ([Giraldo-Calderon et al., 2015](#_ENREF_49)). Red boxes denote tick genes or pathways with genes that are potentially associated with viral infection and identified via RNAi-induced transcript knockdown studies in *Ixodes ricinus* ([Weisheit et al., 2015](#_ENREF_177)) and *I. scapularis* cell lines ([Grabowski et al., 2017a](#_ENREF_52)).


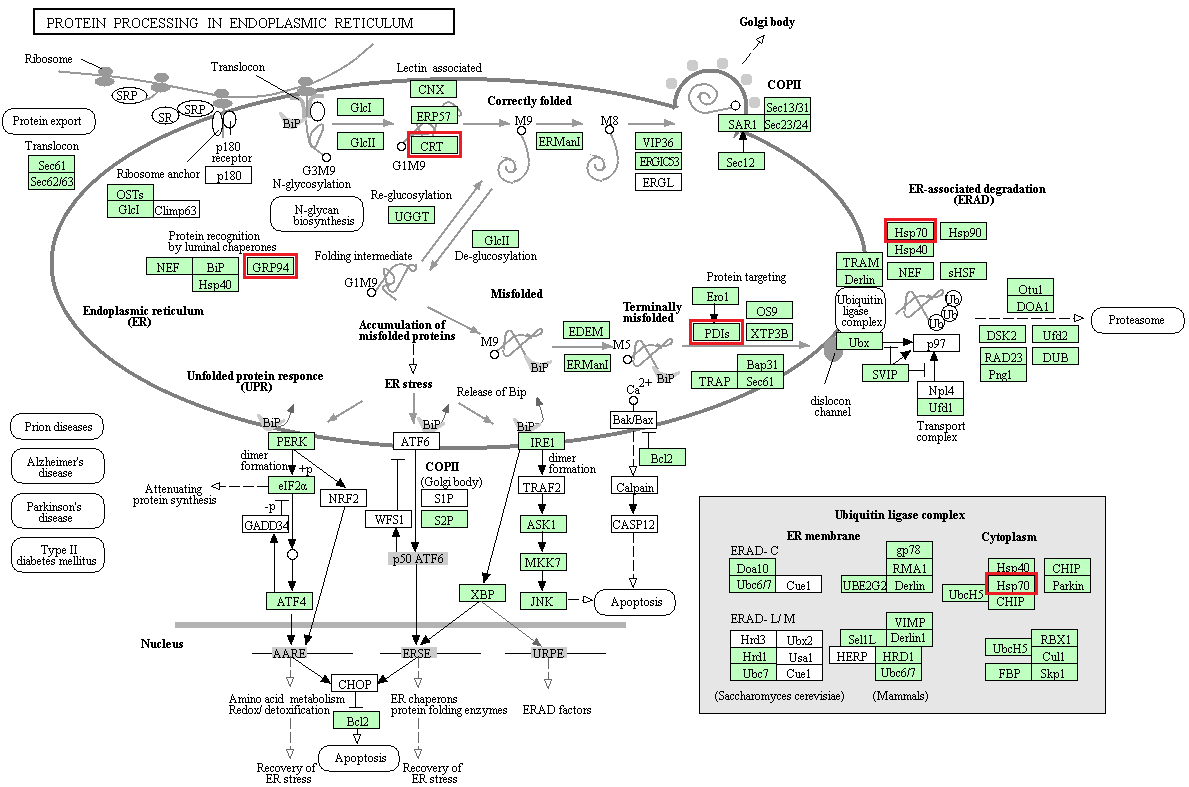


**Figure S2. Proteins mapped to protein processing pathways in the endoplasmic reticulum that may be associated with infection of tick cells by the flavivirus, LGTV.** Boxes denote genes assigned via Kyoto Encyclopedia of Genes and Genomes (KEGG) software ([Kanehisa et al., 2017](#_ENREF_83)) to “protein processing in endoplasmic reticulum”. Green boxes denote orthologous gene models identified from BLAST similarity search of the *Ixodes scapularis* IscaW1 gene model set ([Gulia-Nuss et al., 2016](#_ENREF_61)) downloaded from VectorBase ([Giraldo-Calderon et al., 2015](#_ENREF_49)). Red boxes denote tick genes that are potentially associated with viral infection and identified via RNAi-induced transcript knockdown studies in *Ixodes ricinus* ([Weisheit et al., 2015](#_ENREF_177)) and *I. scapularis* cell lines ([Grabowski et al., 2017a](#_ENREF_52)).

**Table S1. Cellular pathways associated with differential protein expression following flaviviral infection of the tick, mosquito and human cell.**

| **KEGG cellular function and Pathway** | **Host system/Flavivirus** | | |
| --- | --- | --- | --- |
|  | ***Ixodes* spp. ticks/ TBEV & LGTV^1^** | ***Aedes* spp. mosquitoes/**  **DENV^2^** | ***Homo sapiens*/**  **HCV, DENV & JEV^3^** |
| **Genetic information processing** | | | |
| Ribosome | X | X | X |
| Protein processing in ER^4^ | X | X | X |
| Spliceosome | X | X | X |
| RNA transport | X |  | X |
| mRNA surveillance pathway | X |  | X |
| Proteasome | X |  | X |
| RNA degradation | X | X | X |
| Ubiquitin-mediated proteolysis | X |  | X |
| Base excision repair | X |  | X |
| Protein export | X | X | X |
| **Metabolism** | | | |
| Pyruvate metabolism^5^ | X | X | X |
| Citrate cycle (TCA) ^4^ | X | X | X |
| Glyoxylate & dicarboxylate metabolism^4^,^5^ | X | X | X |
| Oxidative phosphorylation | X | X | X |
| Valine, leucine & isoleucine degradation^4^,^5^ | X | X | X |
| Propanoate metabolism^4^,^5^ | X | X | X |
| Glutathione metabolism | X | X | X |
| N-Glycan biosynthesis | X |  | X |
| Aminoacyl-tRNA biosynthesis | X |  | X |
| Lysine degradation^5^ | X | X | X |
| Purine metabolism | X | X | X |
| Pyrimidine metabolism^4^ | X | X | X |
| Fructose and mannose metabolism | X | X | X |
| Alanine, aspartate and glutamate metabolism^4^ | X | X | X |
| D-Glutamine and D-glutamate metabolism | X |  | X |
| **Cellular processes** | | | |
| Phagosome | X | X | X |
| Lysosome | X |  | X |
| Peroxisome | X | X | X |
| **Environmental information processing** | | | |
| mTOR signaling pathway | X | X | X |
| **Organismal systems** | | | |
| Dorso-ventral axis formation | X |  | X |

^1^([Weisheit et al., 2015](#_ENREF_177); [Grabowski et al., 2016](#_ENREF_53))

^2^([Tchankouo-Nguetcheu et al., 2010](#_ENREF_160); [Patramool et al., 2011](#_ENREF_139); [Zhang et al., 2013b](#_ENREF_184); [Chisenhall et al., 2014](#_ENREF_30))

^3^([Pattanakitsakul et al., 2007](#_ENREF_140); [Diamond et al., 2010](#_ENREF_37); [Kanlaya et al., 2010](#_ENREF_84); [Zhang et al., 2013a](#_ENREF_183))

^4^Knockdown of transcripts for protein(s) in this pathway reduced LGTV replication

^5^Knockdown of transcript for protein(s) in this pathway reduced LGTV genome replication
